# Supplementary material for: The Feasibility of Implementing the Flexible Surge Capacity Concept in Bangkok: Willing Participants and Educational Gaps
Source: Int J Environ Res Public Health. 2021 Jul 22;18(15):7793. doi: 10.3390/ijerph18157793 (PMC8345441; doi:10.3390/ijerph18157793)
Supplement: Supplementary file 1 [file ijerph-18-07793-s001.zip › ijerph-1200729-Supplementary A.pdf]

Supplementary A

Information Sheet, Questionnaire and interview guide (English Translation for review only)

Flexible Surge Capacity Questionnaire: In the case of a disaster or an emergency situation, it is extremely critical to utilize the available resources. Moreover, the demand for healthcare services at this critical time is tremendous, and the cooperation between those working in healthcare services and other related organizations is certainly crucial. In order to cooperate effectively, mutual understanding and respect are necessary. The objective of this questionnaire is to find measures to enhance cooperation among the related parties. The respondents are requested to participate on a voluntary basis, and no names of respondents or organizations will be revealed. The questionnaire comprises short questions and will not take a lot of time to respond to.

This research study is a part of a PhD. Thesis that is done as a collaboration between Mahidol University and The University of Gothenburg, Sweden.

Scenario: In the situation that many people are injured and urgently need help, the need for resources may exceed the capacity of the nearby hospitals, leading to cooperation between related organizations and healthcare systems. It is essential for all involved to have knowledge in planning and execution. The researcher aims to understand the ability to cooperate among the health personnel from various fields in specific situations. Please read the given situation and choose the answers that your organization has the potential to carry out.

Situation: There is an accident near your workplace. In total, there are 120 people injured, out of which 40 are severely injured and need urgent help from a big hospital that can perform operations and has an Intensive Care Unit. Another 40 people are moderately injured from inhaling smoke, and it is necessary to monitor their breathing as they may need to be intubated within 2–3 h. Some of these patients may have burns or bone fractures. The last 40 people are slightly injured and can be treated at the hospital or other medical units.

A.1. Primary Care Unit

1. At the time of the incident, if your organization were staffed normally, what help would your organization be able to provide?

   - Be able to provide care for the slightly injured patients
   - Be able to provide primary care for the severely injured patients to stabilize them before transferring them to a larger hospital
   - Be able to provide care for emergency patients not involved in the accident to alleviate the congestion in the emergency room at the hospital
   - Has medical personnel to collaborate with the hospital strengthening the efforts to help the injured.
   - Provide resources: area, medical equipment and devices
   - Perform minor surgery, stitches, or casings for uncomplicated fractures.
   - Provide care for patients
   - Provide psychological care for the injured and the team

- Coordinate the transport of the injured patients back to their homes
- Cannot provide any help
- Others. Please specify ____

2. Things necessary for public health service organizations to be able to provide assistance

- Medical equipment and devices
  Has sufficient medical equipment and devices to assist in special situations
  Lack medical equipment and devices to enhance the ability to provide assistance
  Medical equipment and devices needed to enhance the ability to provide assistance include……
- Local Supplies
  Has adequate supplies to provide assistance in emergency situations
  Lack supplies to enhance the ability to provide assistance
  Supplies needed to enhance the ability to provide assistance include……….
- Personnel
  Knowledge or resources that the personnel need to enhance their ability to provide care for the injured…………
- Supplies of competence

Do you have adequate skills that you would like to have in order to provide assistance in a major accident, when the need for assistance is greater than the available resources?

a. Certainly

b. Yes, but I need additional training

c. No but I can provide assistance if someone provides guidance in …………

d. Others

e. Please provide additional opinion

A.2 Dental Clinic/Veterinary Clinic

1. At the time of the incident, if your organization were staffed normally, what help would your organization be able to provide?

- Be able to provide care for the slightly injured patients
- Be able to provide primary care for the severely injured patients to stabilize them before transferring them to a larger hospital
- Be able to provide care for emergency patients not involved in the accident to alleviate the congestion in the emergency room at the hospital
- Has medical personnel to collaborate with the hospital strengthening the efforts to help the injured.
- Provide resources: area, medical equipment and devices
- Perform minor surgery, stitches, or casings for uncomplicated fractures.
- Provide care for patients
- Provide psychological care for the injured and the team
- Coordinate the transport of the injured patients back to their homes

- Cannot provide any help
- Others. Please specify …………

2. Things necessary for public health service organizations to be able to provide assistance

   - Equipment and devices
     Has sufficient medical equipment and devices to assist in special situations
     Lack medical equipment and devices to enhance the ability to provide assistance
     Medical equipment and de vices needed to enhance the ability to provide assistance include……
   - Local Supplies
     Has adequate supplies to provide assistance in emergency situations
     Lack supplies to enhance the ability to provide assistance
     Supplies needed to enhance the ability to provide assistance include…………
   - Supplies of competence

Do you have adequate skills that you would like to have in order to provide assistance in a major accident, when the need for assistance is greater than the available resources?

a. Certainly

b. Yes, but I need additional training

c. No but I can provide assistance if someone provides guidance in …………

d. Others

e. Please provide additional opinion …………

A.3. Schools/Sports Clubs or Hotels

1. What assistance can you provide for the injured in the case of urgency?

   - Can stop bleeding, repair wounds, perform resuscitation and other emergency procedures
   - Can provide care for the slightly injured
   - Can provide psychological care for patients, who experienced shocks
   - Can provide accommodation for the homeless or the injured
   - Can provide water and food for those in need
   - Can provide care for the children in the case that their adult relatives need to help others
   - Can send staff to help other organizations
   - Cannot provide assistance
   - Others. Please specify…………

2. Do you have adequate skills to provide assistance that you would like to in a major accident?

a. Yes, certainly

b. Yes, but I would like to learn more. Suggestion…………

c. No, but I can provide assistance if I learn relevant skills. Suggestion…………

e. Others…………

3. Necessary items for your organization to be able to provide assistance

   - Equipment and devices

My organization has adequate equipment and devices to provide assistance in emergency situations

My organization lack equipment and devices to enhance the ability provide assistance

In order to enhance ability to provide assistance, the following equipment and devices are required: …………

- Local Supplies

  My organization has adequate facilities to provide assistance in emergency situations

  My organization lack supplies to enhance the ability to provide assistance

  In order to enhance the ability to provide assistance, the following supplies are required:

- Supplies of competence

You have the skills necessary for working in a situation, when a lot of care needs to be provided

a. Yes, certainly.

b. Yes, but I need additional training in …………

c. No, but I can if someone provides advice and training in …………

d. Others

e. Please provide other opinions…………
